# Preoperative Serum Levels of PDGF-AB, PDGF-BB, TGF-α, EGF and ANG-2 in the Diagnosis of Endometrial Cancer

**DOI:** 10.3390/cancers15194815

**Published:** 2023-09-30

**Authors:** Mateusz Kozłowski, Dominika Borzyszkowska, Justyna Mirko, Agnieszka Turoń-Skrzypińska, Katarzyna Piotrowska, Aleksandra Tołoczko-Grabarek, Sebastian Kwiatkowski, Maciej Tarnowski, Iwona Rotter, Aneta Cymbaluk-Płoska

**Affiliations:** 1 Department of Reconstructive Surgery and Gynecological Oncology, Pomeranian Medical University in Szczecin, al. Powstańców Wielkopolskich 72, 70-111 Szczecin, Polandaneta.cymbaluk@gmail.com (A.C.-P.); 2 Department of Medical Rehabilitation and Clinical Physiotherapy, Pomeranian Medical University in Szczecin, Żołnierska 48, 71-210 Szczecin, Poland; 3 Department of Physiology, Pomeranian Medical University in Szczecin, al. Powstancow Wlkp. 72, 70-111 Szczecin, Poland; 4 Department of Genetics and Pathology, Pomeranian Medical University in Szczecin, 71-252 Szczecin, Poland; 5 Department of Obstetrics and Gynecology, Pomeranian Medical University in Szczecin, al. Powstańców Wielkopolskich 72, 70-111 Szczecin, Poland; 6 Department of Physiology in Health Sciences, Faculty of Health Sciences, Pomeranian Medical University in Szczecin, Żołnierska 54, 70-210 Szczecin, Poland

**Keywords:** PDGF-AB, PDGF-BB, TGF-α, EGF, ANG-2, endometrial cancer, diagnosis, diagnostic marker, grow factor, angiogenesis

## Abstract

**Simple Summary:**

In modern, highly industrialized countries, endometrial cancer is the most common gynecological cancer. Endometrial cancer risk has been found to be influenced by a number of variables. The pathogenesis of endometrial cancer is heavily influenced by fatty food consumption in excess, a lack of physical activity and the subsequent development of obesity or diabetes. The first symptoms of endometrial cancer may be unusual vaginal bleeding or pelvic discomfort, but they are also typical of many other diseases. It is important to search for new biomarkers that could help detect endometrial cancer, even if it shows symptoms in its early stages. PDGF-AB, PDGF-BB, TGF-α, EGF and ANG-2 are growth factor proteins. We investigated whether growth factor proteins can be clinically useful diagnostic markers in endometrial cancer. The study’s objective was to identify the clinical relevance of the proteins under investigation. We made a comparison between the serum protein concentrations of people with endometrial cancer and those with non-cancerous endometrial lesions.

**Abstract:**

(1) Background: It is relevant to find new diagnostic biomarkers for endometrial cancer. This study aimed to investigate whether PDGF-AB, PDGF-BB, TGF-α, EGF and ANG-2 could be considered new useful markers for diagnosis and survival of endometrial cancer. (2) Methods: A total of 93 women diagnosed with endometrial cancer (EC) and 66 patients with non-cancerous endometrial lesions (NCEL) were included in this study. (3) Results: Median serum levels of PDGF-AB, PDGF-BB, TGF-α, EGF and ANG-2 were significantly higher in the EC group compared to the NCEL group (for PDGF-AB, PDGF-BB, TGF-α and ANG-2, *p* = 0.0000; for EGF, *p* = 0.0186). The cut-off level of PDGF-AB was set at 127.69 pg/mL with a sensitivity of 87.1% and a specificity of 66.67% (AUC = 0.78, *p* < 0.000001). The cut-off level of PDGF-BB was set at 207.86 ng/L with a sensitivity of 82.8% and a specificity of 75.76% (AUC = 0.85, *p* < 0.000001). The cut-off level of TGF-α was set at 33.85 ng/L with a sensitivity of 82.8% and a specificity of 75.76% (AUC = 0.82, *p* < 0.000001). The cut-off level of EGF was set at 934.76 pg/mL with a sensitivity of 83.87% and a specificity of 28.79% (AUC = 0.61, *p* = 0.018472). The cut-off level of ANG-2 was set at 3120.68 pg/mL with a sensitivity of 72.04% and a specificity of 93.94% (AUC = 0.87, *p* < 0.000001). (4) Conlusion: It was concluded that all the proteins studied could be potential diagnostic markers in endometrial cancer.

## 1. Introduction

In industrialized countries, endometrial cancer (EC) is the most prevalent gynecological cancer [1]. While in 2005 it was observed that about 142,000 women worldwide develop endometrial cancer each year, an article from 2021 already mentions 417,336 cases worldwide each year [2,3]. Most endometrial cancer cases are identified in postmenopausal women, between 65 and 75 years of age [1,3]. One of the most common symptoms is abnormal bleeding from the genital tract, prompting patients to begin diagnostics. Women presenting with abnormal bleeding, which is any postmenopausal reproductive tract bleeding, undergo examinations beginning with transvaginal ultrasound, endometrial biopsy or hysteroscopy, often extended by curettage of the uterine cavity [4]. There is no proof to support screening asymptomatic females for endometrial cancer [5,6,7]. Although endometrial cancer shows symptoms at an early clinical stage of the disease, it seems important to identify biomarkers that would be helpful in the diagnosis of this malignant tumor. To date, a number of factors that increase the risk of endometrial cancer have been distinguished. Obesity plays a huge role in the pathogenesis of endometrial cancer. Compared to women with normal BMI, morbidly obese women had higher mortality rates in a study of women with early endometrial cancer [8]. Risk factors for endometrial cancer include diabetes and hypertension [5]. An active lifestyle, regular physical activity and, consequently, maintaining a healthy weight, a lower risk of diabetes and lower blood pressure helps to lower the risk of endometrial cancer. The use of combined oral contraceptives over a lifetime is also significantly associated with a reduced incidence of endometrial cancer [5,9]. Risk factors such as obesity and diabetes are associated with chronic inflammation. The proteins we examined and described in this study are closely related to angiogenesis [10,11] or the promotion of the inflammatory processes [12,13] or promote cell proliferation and transformation [14,15]. All of these processes are directly related to tumorigenesis. It should be mentioned here that endometrial cancer has a multifactorial etiopathogenesis. Studies show that growth factor proteins play an important role in tumorigenesis [16,17,18,19,20,21,22]. In addition, various studies have appeared in which these proteins have been studied in other gynecological cancers [23,24,25] and in women with abnormal bleeding caused by an IUD [26]. At present, there appear to be no updated studies on PDGF-AB, PDGF-BB, TGF-α, EGF and ANG-2 in endometrial cancer. Therefore, we decided to study the preoperative serum levels of the growth factor proteins PDGF-AB, PDGF-BB, TGF-α, EGF and ANG-2 in the diagnosis of endometrial cancer.

Platelet-derived growth factor (PDGF) is physiologically produced during platelet activation. PDGF has been shown to be a pro-angiogenic factor that plays an important regulatory influence on both normal and diseased blood vessels [10]. There are four various monomeric polypeptide chains: PDGF-A, PDGF-B, PDGF-C and PDGF-D. Through disulfide bonding, they can form five PDGF dimer subtypes including four homodimers and one heterodimer. Our study examined two: heterodimer platelet-derived growth factor AB (PDGF-AB) and homodimer platelet-derived growth factor BB (PDGF-BB). PDGF-AB binds preferably to PDGFRα and PDGF-BB binds to PDGF receptor β (PDGFR-β) [27,28]. It has also been described that PDGF-AB and PDGF-BB similarly contribute to lymph node metastasis and induce tumor lymphatic angiogenesis [29]. Studies conducted on platelet-derived growth factor in relation to endometrial cancer have been sporadic. One study showed that high PDGF-D expression was a poor prognostic factor in endometrial cancer [30]. Patients diagnosed with endometrial cancer who had high PDGF-D levels throughout treatment and strong PDGF-D expression in the primary tumor were more likely to relapse, had a poor prognosis and reduced survival. Therefore, in our study, we focused on other platelet-derived growth factor dimers, PDGF-AB and PDGF-BB, to investigate whether their levels are also important in endometrial cancer. This study will add to the knowledge on these proteins in endometrial cancer.

Epidermal growth factor (EGF) plays a key role in cell differentiation and proliferation [31]. The epidermal growth factor receptor (EGFR) belongs to the same family of receptors (the ERBB family of receptors) that includes the HER2 receptor [32]. This receptor is constantly being explored as a therapeutic target for many cancers. A link has been shown between dysregulated EGF signaling and metabolic reprogramming, especially reprogramming of aerobic glycolysis, which is also known and considered a characteristic of cancer [31]. Regarding endometrial cancer, a study by Gretz et al. observed that EGF stimulates cell growth of a poorly differentiated endometrial adenocarcinoma cell line [33]. However, this is a study from 30 years ago, so the EGF protein and its effect on endometrial cancer requires more recent research.

Transforming growth factor α (TGF-α) is a polypeptide that promotes cell proliferation and transformation [15]. It is also a ligand for the epidermal growth factor receptor, which plays a role in tissue regeneration and bone homeostasis, but unfortunately can promote tumorigenesis [34]. TGF-α expression has previously been studied in other estrogen-dependent cancers such as breast cancer [34]. That study showed that there are significantly higher levels of TGF-α in tissue taken from a metastatic or cancerous lesion compared to healthy tissue.

Another one of the key molecules involved in angiogenesis is angiopoietin 2 (ANG-2) [35]. This molecule disrupts the connections between endothelial and perivascular cells and also promotes apoptosis and regression of blood vessels [11]. ANG-2 is involved in mediating inflammatory processes and is upregulated in many cancers associated with either connected signaling pathways or inflammation [13]. ANG-2 has also been studied in patients with another estrogen-dependent cancers such as breast cancer [36]. Research is already underway on the use of the ANG-2 inhibitor as a therapy against solid tumors, including endometrial cancer [11].

The aim of this study was to determine the clinical significance of the proteins studied. Initially, we compared the serum concentrations of the proteins studied in patients with endometrial cancer (EC) and with non-cancerous endometrial lesion (NCEL). We then determined the correlations between the proteins studied. Finally, the importance of PDGF-AB, PDGF-BB, TGF-α, EGF and ANG-2 in the diagnosis in endometrial cancer was evaluated. Since there are no relevant updated studies, this study will add to the knowledge on these proteins in endometrial cancer.

## 2. Materials and Methods

### 2.1. Study Design

This was an observational study. The study included patients treated for abnormal uterine bleeding. Transvaginal ultrasound was routinely performed in each patient. Patients with a thickened endometrium or suspected endometrial polyps on the transvaginal ultrasound were eligible for this study, and they subsequently underwent hysteroscopy and/or D and C (dilation and curettage). Patients with a histopathological diagnosis of endometrial cancer were then qualified for oncologic surgery. The study group with endometrial cancer and the control group without endometrial cancer were separated. The study group was further divided into subgroups according to histological subtype, grading and clinical stage (staging). Serum levels of the proteins studied were determined in both the study and control groups. Samples were collected between January 2015 and December 2018.

### 2.2. Participants

The premenopausal group included patients with abnormal recurrent uterine bleeding and, on transvaginal ultrasound, a heterogeneous endometrium or shadowing that may correspond to an endometrial polyp (‘P’ in the PALC-COEIN classification of abnormal uterine bleeding). The postmenopausal group included patients whose endometrial thickness on transvaginal ultrasound was 5 mm (in patients not taking hormone replacement therapy) or 8 mm (in patients taking hormone replacement therapy). The exclusion criteria for the study were lack of patient consent, incomplete patient data, history of treatment for another cancer, pelvic inflammatory disease, histological diagnosis of uterine malignancy other than cancer, unbalanced chronic diseases and autoimmune diseases. Ultimately, 159 women were qualified for the study.

At the beginning of the study, the patients’ body mass index (BMI) was measured based on the patients’ weight and height. The BMI was calculated based on the formula BMI = weight (kg)/height^2^ (m^2^). Based on the results, the patients were sorted into two subgroups: with normal weight (BMI 18.5–24.9) and overweight and obese (BMI > 30). Moreover, each of the patients had a blood pressure measurement. Based on the results, we divided the patients into a group with (>140/90) and without hypertension. Moreover, based on the patients’ medical history, we assessed the presence of type 2 diabetes (DM2). The group characteristics are shown in Table 1, Table 2 and Table 3.

### 2.3. Laboratory Analysis

The preoperative serum PDGF-AB, PDGF-BB, TGF-α, EGF and ANG-2 levels were determined using enzyme-linked immunosorbent assays (SunRed Biotechnology, Shanghai, China) according to the manufacturer’s protocol. The specific steps were as follows: the standards of PDGF-AB, PDGF-BB, TGF-α, EGF and ANG-2 were diluted to the corresponding concentration series and the samples and antibodies were added; 50 µL of Streptavidin-HRP was added to each well and incubated for 30 min; the chromogenic agent was used, and the samples were placed in the dark and incubated for 10 min at 37 °C before stop solution was added; the optical density (OD) value of each well at 450 nm was detected using a microplate reader.

### 2.4. Statistical Calculations

Statistical analysis was performed using Statistica 13.3.0 software. The following statistical methods were used to evaluate the collected research material: statistical description, correlation analysis and non-parametric Mann–Whitney U test of significance. A diagnostic test based on the ROC curve was also used. In the first step, statistical description was performed using arithmetic mean, median, minimum and maximum values and asymmetry coefficients. Since the distributions of the quantitative variables were not normal distributions, and the other variables were expressed on a nominal scale, a non-parametric significance test was used to compare the distributions: the Mann–Whitney U test. A nonparametric measure—Spearman’s rank-order correlation coefficient (test of significance for Spearman’s rank-order correlation coefficient)—was also used when testing correlations between quantitative variables. On the other hand, Pearson’s C contingency coefficient based on the χ^2^ statistic (chi square) was used to assess the correlation between variables expressing protein concentrations and variables expressed on a nominal scale. A diagnostic test based on the ROC curve and, in particular, on the area under the ROC curve, which was determined using DeLong’s non-parametric method, was used to distinguish patients with the trait from patients without the trait.

## 3. Results

### 3.1. Comparison of Serum Concentrations of Tested Biomarkers between Groups

We initially determined the serum concentrations of the proteins studied in the EC and NCEL groups. We then compared the median concentrations of the groups. We found that median serum levels of PDGF-AB, PDGF-BB, TGF-α, EGF and ANG-2 were significantly higher in the EC group compared to NCEL (for PDGF-AB, PDGF-BB, TGF-α and ANG-2, *p* = 0.0000; for EGF, *p* = 0.0186). The detailed data are shown in Table 4.

### 3.2. Correlations between Studied Variables

#### 3.2.1. Correlations without Considering Menopausal Status

A statistically significant positive correlation was found between PDGF-AB and PDGF-BB (r = 0.256), PDGF-AB and TGF-α (r = 0.682), PDGF-AB and EGF (r = 0.226), PDGF-AB and ANG-2 (r = 0.473), PDGF-BB and TGF-α (r = 0.373), PDGF-BB and EGF (r = 0.226), PDGF-BB and ANG-2 (r = 0.574), TGF-α and EGF (r = 0.265), TGF-α and ANG-2 (r = 0.548), TGF-α and BMI (r = 0.179) and EGF and ANG-2 (r = 0.344). The detailed data are shown in Table 5. 

#### 3.2.2. Correlations between Studied Proteins in Premenopausal Patients

In the next step, the correlations between the proteins studied were examined in the group containing premenopausal patients. A statistically significant positive correlation was found between PDGF-AB and PDGF-BB (r = 0.344), PDGF-AB and TGF-α (r = 0.704), PDGF-AB and ANG-2 (r = 0.587), PDGF-BB and TGF-α (r = 0.425), PDGF-BB and ANG-2 (r = 0.419), TGF-α and ANG-2 (r = 0.714) and EGF and ANG-2 (r = 0.31). The detailed data are shown in Table 6.

#### 3.2.3. Correlations between Studied Proteins in Postmenopausal Patients

In the subsequent step, the correlations between the proteins studied were examined in the group containing postmenopausal patients. A statistically significant positive correlation was found between PDGF-AB and PDGF-BB (r = 0.23), PDGF-AB and TGF-α (r = 0.671), PDGF-AB and EGF (r = 0.225), PDGF-AB and ANG-2 (r = 0.428), PDGF-BB and TGF-α (r = 0.356), PDGF-BB and EGF (r = 0.241), PDGF-BB and ANG-2 (r = 0.63), TGF-α and EGF (r = 0.264), TGF-α and ANG-2 (r = 0.47) and EGF and ANG-2 (r = 0.358). The detailed data are shown in Table 7.

### 3.3. Receiver Operating Characteristic (ROC) Curve for Using PDGF-AB, PDGF-BB, TGF-α, EGF and ANG-2 to Distinguish between Endometrial Cancer and Non-Cancerous Endometrial Lesions

#### 3.3.1. ROC without Considering Menopausal Status

**Figure 1:**
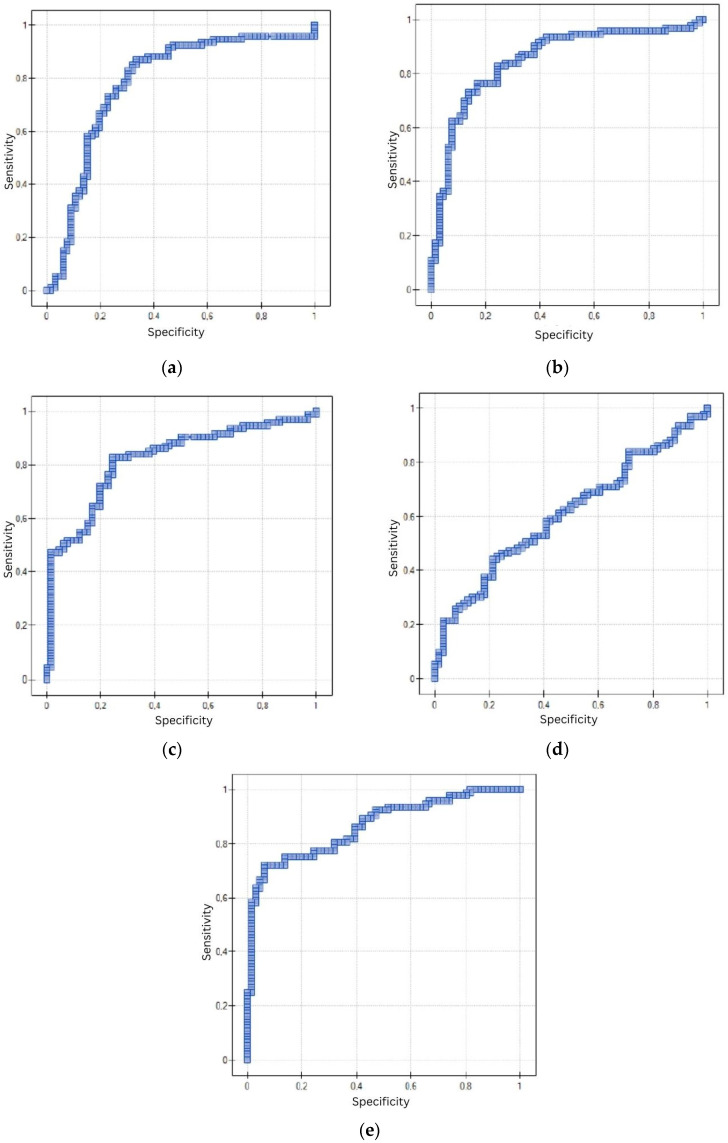
ROC curve using PDGF-AB (**a**), PDGF-BB (**b**), TGF-α (**c**), EGF (**d**) and ANG-2 (**e**) to distinguish between EC and NCEL without considering menopausal status.

The diagnostic significance of the tested proteins was evaluated between the study group (EC) and the control group (NCEL). The cut-off level of PDGF-AB was set at 127.69 pg/mL with a sensitivity of 87.1% and a specificity of 66.67% (AUC = 0.78, *p* < 0.000001). The cut-off level of PDGF-BB was set at 207.86 ng/L with a sensitivity of 82.8% and a specificity of 75.76% (AUC = 0.85, *p* < 0.000001). The cut-off level of TGF-α was set at 33.85 ng/L with a sensitivity of 82.8% and a specificity of 75.76% (AUC = 0.82, *p* < 0.000001). The cut-off level of EGF was set at 934.76 pg/mL with a sensitivity of 83.87% and a specificity of 28.79% (AUC = 0.61, *p* = 0.018472). The cut-off level of ANG-2 was set at 3120.68 pg/mL with a sensitivity of 72.04% and a specificity of 93.94% (AUC = 0.87, *p* < 0.000001). The detailed data are shown in Table 8 and Figure 1.

#### 3.3.2. Receiver Operating Characteristic (ROC) Curve for Using PDGF-AB, PDGF-BB, TGF-α, EGF and ANG-2 to Distinguish between Endometrial Cancer and Non-Cancerous Endometrial Lesions in Premenopausal Patients

**Figure 2:**
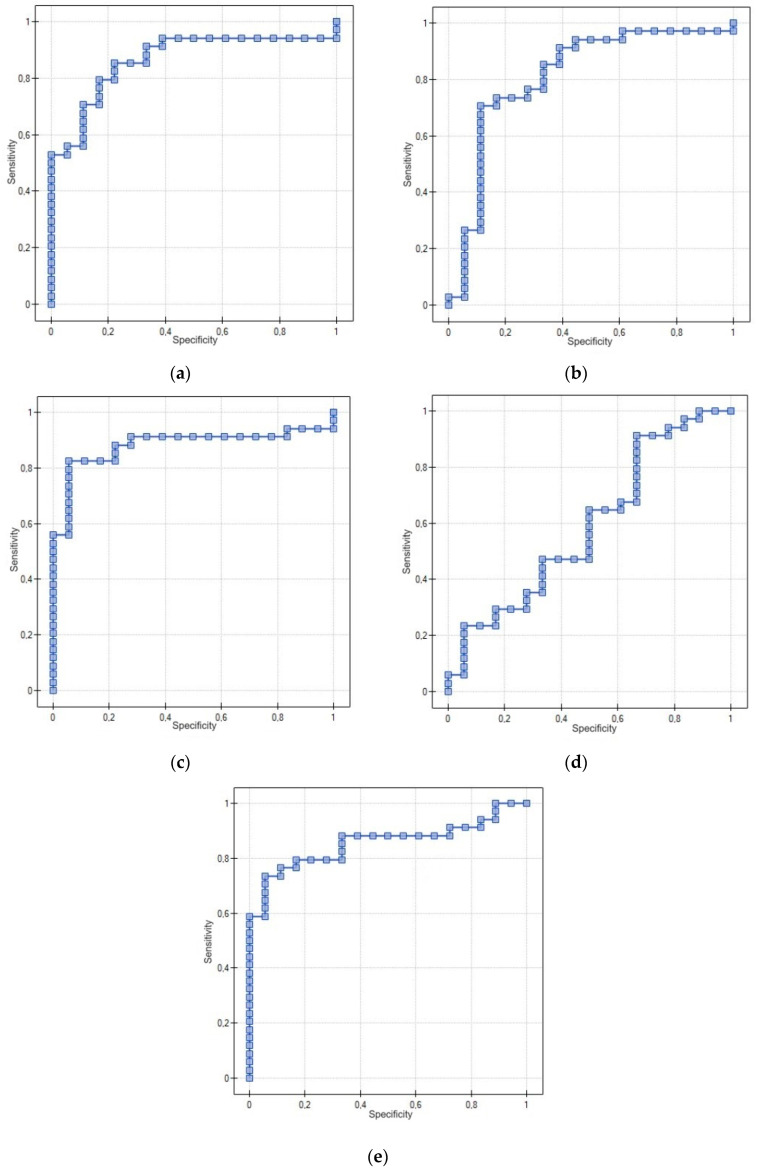
ROC curve using PDGF-AB (**a**), PDGF-BB (**b**), TGF-α (**c**), EGF (**d**) and ANG-2 (**e**) to distinguish between endometrial cancer and non-cancerous endometrial lesions in premenopausal patients.

The diagnostic significance of the tested proteins was evaluated between the study group (EC) and the control group (NCEL) in premenopausal patients. The cut-off level of PDGF-AB was set at 106.66 pg/mL with a sensitivity of 94.12% and a specificity of 61.11% (AUC = 0.86, *p* = 0.000018). The cut-off level of PDGF-BB was set at 197.79 ng/L with a sensitivity of 94.12% and a specificity of 55.56% (AUC = 0.81, *p* = 0.000239). The cut-off level of TGF-α was set at 34.71 ng/L with a sensitivity of 82.35% and a specificity of 94.44% (AUC = 0.88, *p* = 0.000007). The cut-off level of EGF was set at 934.76 pg/mL with a sensitivity of 91.18% and a specificity of 33.33% (AUC = 0.59, *p* = 0.298966). The cut-off level of ANG-2 was set at 3132.47 pg/mL with a sensitivity of 73.53% and a specificity of 94.44% (AUC = 0.86, *p* = 0.000028). The detailed data are shown in Table 9 and Figure 2.

#### 3.3.3. Receiver Operating Characteristic (ROC) Curve for Using PDGF-AB, PDGF-BB, TGF-α, EGF and ANG-2 to Distinguish between Endometrial Cancer and Non-Cancerous Endometrial Lesions in Postmenopausal Patients

**Figure 3:**
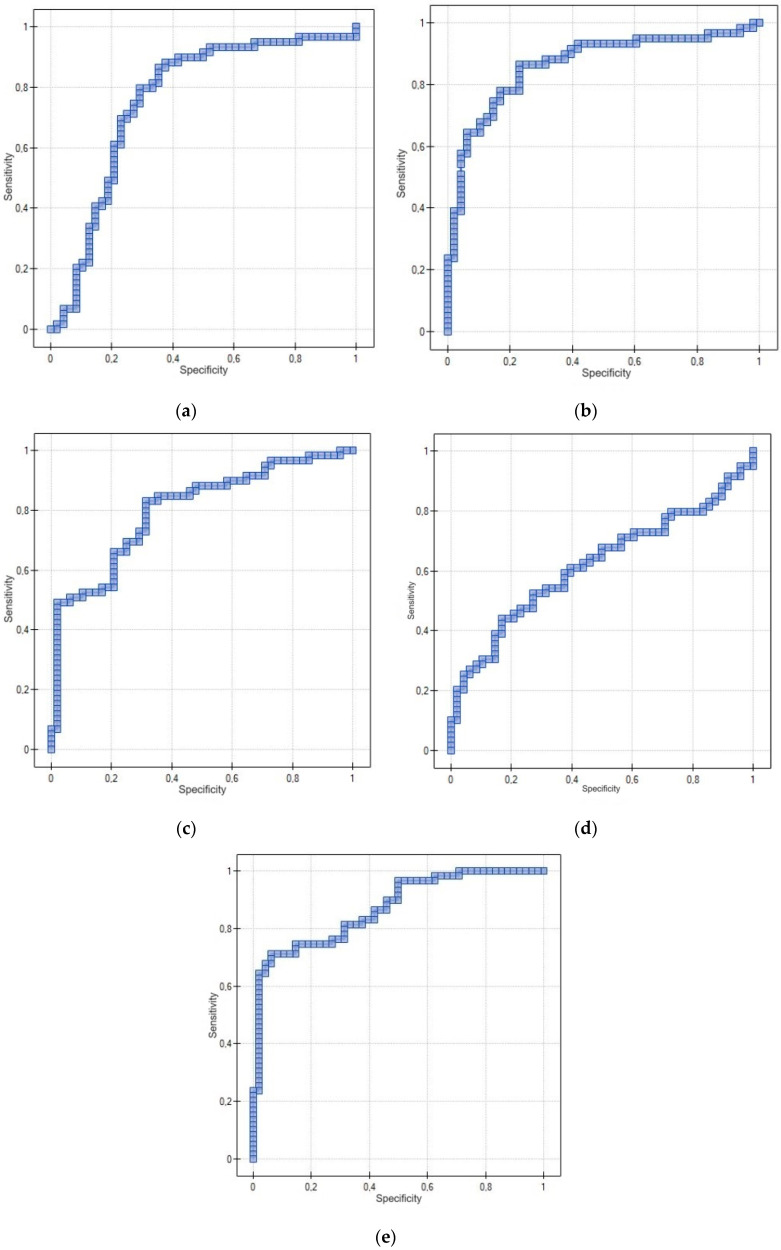
ROC curve using PDGF-AB (**a**), PDGF-BB (**b**), TGF-α (**c**), EGF (**d**) and ANG-2 (**e**) to distinguish between endometrial cancer and non-cancerous endometrial lesions in postmenopausal patients.

The diagnostic significance of the tested proteins was evaluated between the study group (EC) and the control group (NCEL) in postmenopausal patients. The cut-off level of PDGF-AB was set at 130.26 pg/mL with a sensitivity of 86.44% and a specificity of 64.58% (AUC = 0.76, *p* = 0.000005). The cut-off level of PDGF-BB was set at 207.86 ng/L with a sensitivity of 86.44% and a specificity of 77.08% (AUC = 0.86, *p* < 0.000001). The cut-off level of TGF-α was set at 33.85 ng/L with a sensitivity of 83.05% and a specificity of 68.75% (AUC = 0.8, *p* < 0.000001). The cut-off level of EGF was set at 1231.72 pg/mL with a sensitivity of 52.54% and a specificity of 72.92% (AUC = 0.62, *p* = 0.032685). The cut-off level of ANG-2 was set at 3120.68 pg/mL with a sensitivity of 71.19% and a specificity of 93.75% (AUC = 0.87, *p* < 0.000001). The detailed data are shown in Table 10 and Figure 3.

## 4. Discussion

Although endometrial cancer is one of the most common gynecological cancers and its morbidity rate is increasing, it is still not yet as investigated as ovarian or cervical cancer. There is currently no biomarker that is used for diagnostic purposes in endometrial cancer, but that is the reason why it is so important to keep conducting research. Our study was conducted to answer the question if PDGF-AB, PDGF-BB, TGF-α, EGF and ANG-2 could be useful as markers to diagnose patients with endometrial cancer. According to the results, the median serum levels of PDGF-AB, PDGF-BB, TGF-α, EGF and ANG-2 were significantly higher in the group of patients that suffered from EC compared to patients with NCEL. There seemed to be an association between the serum concentrations of the proteins tested in our study. One of the limitations of our study was the small number of patients in both the study and control groups. Another limitation was that the control group consisted of both patients who had endometrial polyps and patients who had a thickened endometrium. However, this was due to the division into patients having non-cancerous lesions, which included endometrial polyps and thickened endometrium, and those with cancerous lesions. In this way, we clearly delineated the study group and the control group.

PDGF has been shown to be a pro-angiogenic factor, but not many studies have considered its dimer subtypes. In the literature, we can find references to other types of cancers than the one tested during our study, such as ovarian cancer [18,19], breast cancer [21], pleural mesothelioma [37], colorectal cancer [38] or fibrosarcoma [29]. Horala et al. conducted a study that focused on the value of angiogenic markers in the serum of patients with ovarian cancer. They conducted their study on patients suffering from OC, borderline ovarian tumors and benign ovarian tumors. The aim of the study was to establish a multimarker model based on several biomarkers that would have the best outcome in diagnosing malignant versus benign tumors and that would also include platelet-derived growth factor AB/BB [18]. However, these results contradict the claims of a study that focused on lung cancer, where the serum levels of PDGF-AA and PDGF-AB/BB were lower in patients with non-small cell lung cancer and with small cell lung cancer compared to healthy controls [39]. Although PDGF plays an important role in growth and development of the vessels in a healthy tissue, it also can contribute to lymphatic metastases and cancer evasion of the anti-VEGF treatment [40]. A study by Cao et al. on a mouse model investigated how PDGF-BB affects lymphatic metastasis formation in murine fibrosarcoma. In vitro, PDGF-BB stimulated the MAP kinase activity of lymphatic endothelial cells, while in vivo it strongly induced lymphatic vessel growth. Therefore, its expression would also stimulate lymphangiogenesis, which could lead to a higher risk of lymph node metastasis [29]. Sun et al. studied TGF-α expression in breast cancer and its bone metastases. They observed TGF-α in much higher levels in carcinomas compared to the healthy patients; but not in the serum of the patients, but in the tissue collected from bone metastases in breast cancer, the primary lesion and healthy tissue [34]. According to that study, the level of TGF-α in the affected tissues in bone metastatic breast cancer was the highest, but also was at a higher level in non-BM-BC than in the benign group. 

Attention to biomarker concentrations and their changes with cancer treatment also seems to be an important topic. The literature provides general information on changes in PDGF levels in surgically treated colorectal cancer [41] and in the treatment of recurrent or persistent epithelial ovarian cancer [42]. In colorectal cancer, it was observed that PDGF-AB levels in intraoperatively collected blood in stage G3 colorectal cancer were significantly higher than in stage G2 [41]. A study in ovarian cancer examined that higher plasma levels of PDGF-AB, PDGF-BB and VEGF before treatment were associated with shorter progression-free survival and overall survival [42]. During our study, we did not include follow-up of our patients after EC treatment or in case of relapse, as this was not the purpose of our study. Instead, it seems worthy of being taken into account in conducting further studies.

Among premenopausal women, the incidence of endometrial cancer is in the range of about 14–20% [43], also in young patients who did not yet have children. Patients who are still menstruating may not notice abnormal bleeding as easily as those who are already postmenopausal. And this is an alarming symptom, prompting further diagnosis. That is why finding new ways of diagnosing EC is crucial. The goal is to find a method that would be non-invasive and that would allow medical professionals to diagnose patients in early stages and therefore to help young patients to maintain their fertility. As endometrial cancer affects the organ that is crucial to reproduction, it is very important to find treatment methods that would be as safe for the reproductive system as possible. The gold standard treatment for early stage endometrial cancer is now hysterectomy compared with bilateral salpingo-oophorectomy and lymphadenectomy. In the literature, we can find a systematic review that collected the most promising studies on methods of management for patients with early stage EC—both conservative and radical methods [44]. In this study, according to the results it is possible to implement conservative treatment techniques to allow pregnancy but it should be a temporary solution. The methods that were the most promising include using hysteroscopic resectoscope combined with LNG-IUD or medroxyprogesterone acetate. However, though fertility-sparing treatment methods appear to show encouraging results, they are not yet tested enough to be considered as a standard treatment. 

The results of the presented research seem promising. However, attention should be paid to the use of proteins in the diagnosis of EC without and with consideration of menopausal status. Particularly relevant is the use of the EGF protein in the diagnosis of EC in a group of premenopausal patients, in which a non-significant result was obtained here. In light of currently available studies, it is difficult to explain this result, especially since this protein is similar in function to the other proteins studied here. The diagnostic utility of this protein in a group of postmenopausal patients should also be interpreted with great caution, as the sensitivity is 52.54%—a significantly lower sensitivity compared to the other proteins. The other proteins show significantly similar sensitivity and specificity in both premenopausal and postmenopausal patients.

As we mentioned earlier, the data collected during our study are limited by the size of our study group. For a confirmation of the results, it would be necessary to conduct a similar study again in the future, but with a larger group of patients. The data gathered in the literature confirm that the elevated serum concentration levels of proteins tested in our study are found in many other carcinomas apart from endometrial cancer. 

## 5. Conclusions

These results suggest that all of the proteins studied could be potential biomarkers in the diagnosis of endometrial cancer. However, taking into account the menopausal status of the patients, in the group of premenopausal patients, PDGF-AB, PDGF-BB, TGF-α and ANG-2 could be potential biomarkers for the diagnosis of endometrial cancer. In the group of postmenopausal patients, as in the total cohort, all the proteins tested could be markers of endometrial cancer. In order to confirm the results, it would be necessary to conduct a similar study again, especially involving a larger group of female patients.

## Figures and Tables

**Table 1:** Characteristics of patients including endometrial cancer characteristics.

Characteristics	Number of Patients (%)
Endometrial cancer	
Yes	93 (58)
No	66 (42)
Endometrial cancer	
Type 1	82 (88)
Type 2	11 (12)
Clinical staging	
FIGO I and II	88 (95)
FIGO III and IV	5 (5)
Histopathological grading	
Grade 1	37 (40)
Grade 2	34 (37)
Grade 3	22 (24)

**Table 2:** Characteristics of patients including clinical data.

Clinical and Demographic Characteristics	Total Cohort (*n* = 159)	Endometrial Cancer (*n* = 93)	NCEL (*n* = 66)	*p*-Value
Median (IQR)				
Age (years old)	55 (43–67)	51 (43–63)	59.5 (43–71)	0.1106
BMI (kg/m^2^)	27.9 (24.2–31.7)	28.6 (25.4–31.7)	27 (23–31)	0.0397
Number (%)				
Age (years old)				
<65	114 (72)	74 (80)	40 (61)	0.0967
≥65	45 (28)	19 (20)	26 (39)	0.2889
BMI				
<25	43 (27)	22 (24)	21 (32)	0.5922
≥25	116 (73)	71 (76)	45 (68)	0.1176
Menopausal status				
Premenopausal	52 (33)	34 (37)	18 (27)	0.0387
Postmenopausal	107 (67)	59 (63)	48 (73)	0.3035
Hypertension				
Yes	82 (52)	49 (53)	33 (50)	0.3600
No	77 (48)	44 (47)	33 (50)	0.5807
Diabetes mellitus				
Yes	73 (46)	42 (45)	31 (47)	0.5577
No	86 (54)	51 (55)	35 (53)	0.3123

**Table 3:** Characteristics representing age in a group of premenopausal and postmenopausal women.

Characteristics		Total Cohort
Median (IQR)		
Age (years old)	Premenopausal	37 (32.5–43)
	Postmenopausal	62 (54–71)

**Table 4:** Comparison of concentrations of studied proteins between patients with endometrial cancer and non-cancerous endometrial lesions.

Characteristics		EC	NCEL	*p*-Value
PDGF-AB (pg/mL)	Median	173.71	118.23	0.0000
	Q1–Q3	140.53–198.11	96.79–142.89	
PDGF-BB (ng/L)	Median	250.98	185.33	0.0000
	Q1–Q3	213.97–298.61	160.43–207.43	
TGF-α (ng/L)	Median	41.03	30.38	0.0000
	Q1–Q3	35.01–47.45	26.79–33.66	
EGF (pg/mL)	Median	1231.72	1147.96	0.0186
	Q1–Q3	1028.56–1374.82	923.46–1266.39	
ANG-2 (pg/mL)	Median	3649.26	2306.98	0.0000
	Q1–Q3	3011.34–4512.63	1811.25–2917.67	

EC—endometrial cancer group; NCEL—non-cancerous endometrial lesions.

**Table 5:** The correlations between PDGF-AB, PDGF-BB, TGF-α, EGF, ANG-2, age and BMI, presented as the Spearman’s ranges‚ rs correlation coefficients.

Variable		PDGF-AB	PDGF-BB	TGF-α	EGF	ANG-2
PDGF-AB	rs	1	0.256	0.682	0.226	0.473
	*p*-Value	-	0.00114	0.0000001	0.00425	0.0000001
PDGF-BB	rs	0.256	1	0.373	0.226	0.574
	*p*-Value	0.00114	-	0.0000001	0.004216	0.0000001
TGF-α	rs	0.682	0.373	1	0.265	0.548
	*p*-Value	0.0000001	0.0000001	-	0.000748	0.0000001
EGF	rs	0.226	0.226	0.265	1	0.344
	*p*-Value	0.00425	0.004216	0.000748	-	0.000009
ANG-2	rs	0.473	0.574	0.548	0.344	1
	*p*-Value	0.0000001	0.0000001	0.0000001	0.000009	-

**Table 6:** The correlations between PDGF-AB, PDGF-BB, TGF-α, EGF and ANG-2 in premenopausal patients, presented as the Spearman’s ranges‚ rs correlation coefficient.

Variable		PDGF-AB	PDGF-BB	TGF-α	EGF	ANG-2
PDGF-AB	rs	1	0.344	0.704	0.265	0.587
	*p*-Value	-	0.012623	0.0000001	0.057364	0.000005
PDGF-BB	rs	0.344	1	0.425	0.214	0.419
	*p*-Value	0.012623	-	0.001694	0.12729	0.002018
TGF-α	rs	0.704	0.425	1	0.259	0.714
	*p*-Value	0.0000001	0.001694	-	0.063521	0.0000001
EGF	rs	0.265	0.214	0.259	1	0.31
	*p*-Value	0.057364	0.12729	0.063521	-	0.02549
ANG-2	rs	0.587	0.419	0.714	0.31	1
	*p*-Value	0.000005	0.002018	0.0000001	0.02549	-

**Table 7:** The correlations between PDGF-AB, PDGF-BB, TGF-α, EGF and ANG-2 in postmenopausal patients, presented as the Spearman’s ranges‚ rs correlation coefficient.

Variable		PDGF-AB	PDGF-BB	TGF-α	EGF	ANG-2
PDGF-AB	rs	1	0.23	0.671	0.225	0.428
	*p*-Value	-	0.017217	0.000000	0.020006	0.000004
PDGF-BB	rs	0.23	1	0.356	0.241	0.63
	*p*-Value	0.017217	-	0.000169	0.012535	0.000000
TGF-α	rs	0.671	0.356	1	0.264	0.47
	*p*-Value	0.000000	0.000169	-	0.005945	0.000000
EGF	rs	0.225	0.241	0.264	1	0.358
	*p*-Value	0.020006	0.012535	0.005945	-	0.000153
ANG-2	rs	0.428	0.63	0.47	0.358	1
	*p*-Value	0.000004	0.000000	0.000000	0.000153	-

**Table 8:** Diagnostic values of studied proteins for patients with endometrial cancer without considering menopausal status.

Marker	AUC (95% CI)	Sensitivity (%)	Specificity (%)	PPV (%)	NPV (%)	*p*-Value	Cut-Off Value
PDGF-AB	0.78 (0.7–0.86)	87.1	66.67	78.64	78.57	<0.000001	127.69 pg/mL
PDGF-BB	0.85 (0.79–0.91)	82.8	75.76	82.8	75.76	<0.000001	207.86 ng/L
TGF-α	0.82 (0.75–0.89)	82.8	75.76	82.8	75.76	<0.000001	33.85 ng/L
EGF	0.61 (0.52–0.7)	83.87	28.79	62.4	55.89	0.018472	934.76 pg/mL
ANG-2	0.87 (0.81–0.92)	72.04	93.94	94.37	70.45	<0.000001	3120.68 pg/mL

**Table 9:** Diagnostic values of studied proteins for patients with endometrial cancer in premenopausal patients.

Marker	AUC (95% CI)	Sensitivity (%)	Specificity (%)	PPV (%)	NPV (%)	*p*-Value	Cut-Off Value
PDGF-AB	0.86 (0.76–0.97)	94.12	61.11	82.05	84.62	0.000018	106.66 pg/mL
PDGF-BB	0.81 (0.67–0.95)	94.12	55.56	80	83.33	0.000239	197.79 ng/L
TGF-α	0.88 (0.78–0.98)	82.35	94.44	96.55	73.91	0.000007	34.71 ng/L
EGF	0.59 (0.42–0.76)	91.18	33.33	72.09	66.67	0.298966	934.76 pg/mL
ANG-2	0.86 (0.75–0.96)	73.53	94.44	96.15	65.38	0.000028	3132.47 pg/mL

**Table 10:** Diagnostic values of studied proteins for patients with endometrial cancer in postmenopausal patients.

Marker	AUC (95% CI)	Sensitivity (%)	Specificity (%)	PPV (%)	NPV (%)	*p*-Value	Cut-Off Value
PDGF-AB	0.76 (0.66–0.86)	86.44	64.58	75	79.49	0.000005	130.26 pg/mL
PDGF-BB	0.86 (0.79–0.94)	86.44	77.08	82.26	82.22	<0.000001	207.86 ng/L
TGF-α	0.8 (0.72–0.88)	83.05	68.75	76.56	76.74	<0.000001	33.85 ng/L
EGF	0.62 (0.51–0.73)	52.54	72.92	70.45	55.56	0.032685	1231.72 pg/mL
ANG-2	0.87 (0.8–0.94)	71.19	93.75	93.33	72.58	<0.000001	3120.68 pg/mL

## Data Availability

The data presented in this study are available from the corresponding author, D.B., upon reasonable request.
